# Selective Homocysteine Assay with Cucurbit[7]uril by pH Regulation

**DOI:** 10.4014/jmb.2201.01016

**Published:** 2022-01-23

**Authors:** Won-Bin Bae, Hee-Joon Kim, Kwang-Hwan Jhee

**Affiliations:** Department of Applied Chemistry, Kumoh National Institute of Technology, Gumi 39177, Republic of Korea

**Keywords:** Homocysteine, cysteine, cucurbit[7]uril, homocystine, pH effect, supramolecular complexation

## Abstract

We report the effect of pH on the supramolecular complexation of two biothiols, viz., homocysteine (Hcy) and cysteine (Cys), with cucurbit[7]uril (CB[7]). Under basic pH conditions, Cys did not complex with CB[7], whereas Hcy efficiently complexed with CB[7], as confirmed by ^1^H NMR spectroscopy and Ellman’s reagent (5,5’-dithio-bis(2-nitrobenzoic acid), DTNB) assay. ^1^H NMR and Raman spectroscopic studies revealed that, in the absence of CB[7], Hcy auto-oxidized slowly (~36 h) to homocystine (HSSH) under basic pH conditions. However, the rate of Hcy oxidation increased by up to 150 fold in the presence of CB[7], as suggested by the DTNB assay. Thus, supramolecular complexation under basic pH conditions led to the formation of a HSSH-CB[7] complex, and not Hcy-CB[7]. The results indicate that Hcy is rapidly oxidized to HSSH under the catalysis of CB[7], which acts as a reaction chamber, in basic pH conditions. Our studies suggest that Hcy concentration, a risk factor for cardiovascular disease, can be selectively and more easily quantified by supramolecular complexation with CB [7].

## Introduction

Biothiols are a class of substances that play a vital role in the maintenance of life by means of an equilibrium that exists between their reduced free thiol and oxidized disulfide forms in biological systems [1]. The intracellular biothiols, cysteine (Cys) and homocysteine (Hcy) are closely linked to physiological and pathological processes in complex biological environments [2]. For example, the Hcy concentration in plasma or serum is associated with complications of pregnancy, mental disorders, neural tube defects, and cognitive impairment in the elderly [3]. Furthermore, results from clinical and pathological studies have provided sufficient evidence that an elevated Hcy concentration is a cardiovascular risk factor in the general population [4-7]. Owing to the high degree of similarity in both the structure and chemical properties between Cys and Hcy under physiological conditions, selective Hcy detection is quite difficult but important for the early diagnosis and treatment of several related diseases. To date, many analytical methods for Hcy and Cys have been developed in conjunction with high-performance liquid chromatography, capillary electrophoresis, electrochemical detection, UV-visible spectroscopy, fluorescence spectroscopy, and enzymatic assays [8-15]. Among these, fluorescence spectroscopy is generally employed for Hcy detection because of its high sensitivity, facile operation, visual signal transduction, and suitability for in vivo analysis. Moreover, most methods are time and cost intensive.

Molecular recognition and sensing of biological molecules in aqueous solutions have many applications in diverse fields such as drug delivery, nutrition analysis, biochip development, and disease diagnosis [16, 17]. Cucurbit[*n*]uril (CB[*n*]), where *n* indicates the number of glycoluril units (*n* = 5–8, 10), is an interesting supramolecule composed of glycoluril (=C_4_H_2_N_4_O_2_=) monomers linked by methylene (-CH_2_) bridges. All CB[*n*] structures have the same cavity depth, but the cavity size differs according to *n* [15]. Moreover, CB[*n*] molecules have specific binding properties for small biomolecules, peptides, and proteins depending on the cavity size [15, 18-23]. Previously, we studied the supramolecular complexation of Hcy and Cys with CB[7] in neutral aqueous solutions [15], and reported three Hcy assay methods based on the host–guest complexation of Hcy with CB[7]. According to our studies, the complexation ratio between Hcy and CB[7] converges to 50% saturation [7]. As the pK_a_ values of Cys and Hcy are different (8.33 for Cys [24] and 8.87 for Hcy [25]), the propensity of Cys and Hcy ionization or oxidation is sensitive to local variations in the pH. In this study, we investigated the ratio and rate of supramolecular complexation of Hcy with CB[7] according to the changes in the pH using a 5,5’-dithio-bis(2-nitrobenzoic acid) (DTNB) assay, ^1^H NMR spectroscopy, including the COSY technique, and Raman spectroscopy.

## Materials and Methods

### Reagents

Cys, cystine (CSSC), Hcy, homocystine (HSSH), DTNB, CB[7], iodoacetamide, ammonium acetate (C_2_H_7_NO_2_), Tris-HCl buffer (pH 8.8), and deuterium oxide (D_2_O) were purchased from Sigma-Aldrich Chemicals (USA). Hydrochloric acid (HCl) and sodium bicarbonate (NaHCO_3_) were purchased from Duksan Pure Chemical Co., Ltd. (Korea). Potassium dihydrogen phosphate (KH_2_PO_4_), dipotassium hydrogen phosphate (K_2_HPO_4_), sodium dihydrogen phosphate (NaH_2_PO_4_), and disodium hydrogen phosphate (Na_2_HPO_4_) were purchased from Samchun Pure Chemical Co., Ltd. (Korea). Sodium chloride (NaCl), ammonia solution (NH_4_OH), acetic acid (CH_3_COOH), and sodium carbonate (Na_2_CO_3_) were purchased from Daejung Chemicals & Metals Co., Ltd.(Korea).

### Chemical Detection of Cys and Hcy Using the DTNB Assay

The Cys-CB[7] and Hcy-CB[7] binding ratios and the changes in the absorbances of Cys and Hcy solutions with the incubation time with CB[7] were evaluated using a DTNB colorimetric assay. DTNB, known as the Ellman’s reagent, can be used for analyzing low- molecular-mass thiols in both pure solutions and biological samples; it is a reliable method for determining the amounts of Cys, Hcy, and other thiols in a solution. Stock solutions of 5 mM Cys, 5 mM Hcy, 2 mM CB[7], and 2 mM DTNB were prepared in 100 mM sodium phosphate buffer (pH 6.0–7.0), 100 mM PBS buffer (pH 7.5), 100 mM borate buffer (pH 8.0–8.5), and 100 mM Tris-HCl buffer (pH 8.8), respectively. To determine the Cys-CB[7] and Hcy-CB[7] binding ratios, 10 μl volumes of 500 μM Cys (5 nmol) and 500 μM Hcy (5 nmol) were reacted separately with 50 μl of CB[7] (100 nm) for 5 min. Then, each sample was mixed with 120 μl of the DTNB solution. Another batch of Cys/CB[7] or Hcy/CB[7] samples was reacted with iodoacetamide at pH 6.0 and 8.8, and then mixed with 120 μl of the DTNB solution. The absorbances of the resulting solutions were measured at 412 nm using a spectrophotometer (Agilent 8453, USA) after 3 min of reaction with DTNB. Iodoacetamide (100 nmol) is a thiol-blocking electrophilic reagent that was used as a negative control.

The time-dependent changes in the Cys and Hcy solutions without CB[7] were also studied using the DTNB assay under different pH conditions for the analysis of autoxidation. Cys (500 μM) and Hcy solutions (500 μM) were incubated at 30°C for 3, 6, 9, 12, 18, 24, and 36 h under pH 6.0 and 8.8 conditions. Thereafter, 10 μl of the incubated 500 μM Cys or 500 μM Hcy solution was mixed with 70 μl of the sodium phosphate buffer (pH 6.0) and Tris-HCl (pH 8.8), respectively. Subsequently, the resulting solution was reacted with 120 μl of 2 mM DTNB for 3 min, and then the absorbance at 412 nm was recorded. All the experiments were performed at 25°C.

The data were analyzed with SPSS version 28.0.1.0 0 (SPSS Inc., USA) using one-way ANOVA and post-hoc comparisons by the Tukey test. Data graphing was presented by utilizing Origin 2021b (OriginLab Corporation, USA) and the results were presented as mean ± SEM. *p* < 0.05 was considered statistically significant.

### ^1^H NMR Spectroscopy

The 1D and 2D NMR (COSY) spectra of all the samples were recorded at 298 K using a Bruker Advance III 400 spectrophotometer. The resonance frequency was 400.13 MHz, and the spectral width was 9.9 ppm. The relaxation delay time, D1, was set as 1.0 s. All samples were incubated at 25°C for 36 h. NMR measurements were made by reacting 0.2 mM Cys, 0.2 mM CSSC, 1.0 mM Hcy, and 0.5 mM HSSH with 10–20 equivalents of CB[7] in 5 mM sodium phosphate (pH 6.0) and 5 mM carbonate-bicarbonate buffer (pH10.0) in D_2_O. Other acquisition parameters were as follows: P1, 12 μs; time domain, 65 K; and eight scans.

### Raman Spectroscopy

Raman spectra were recorded using 785 nm radiation from a diode laser over the range of 100–3,200 cm^−1^. Cys, CSSC, Hcy, and HSSH powders were used as controls. The Raman spectra were acquired using powders obtained by incubating 50 mM Cys and Hcy at pH 6.0 and 10.0, respectively, for 36 h and then drying the samples using a centrifugal vacuum concentrator (Ecospin 3180C, Korea).

## Results

### Determination of the Hcy-CB[7] Complexation Ratio Using the DTNB Assay

**Fig. 1:**
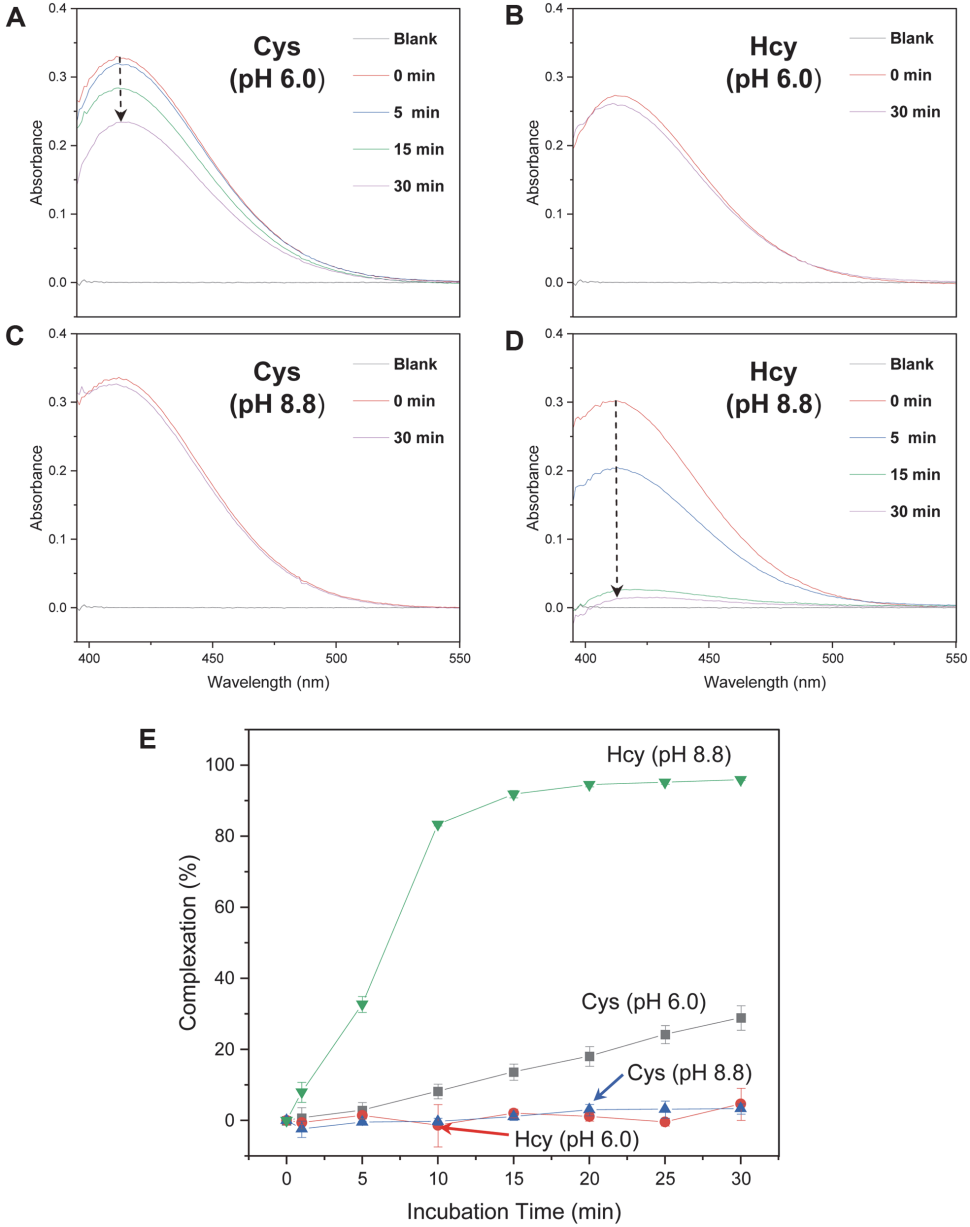
Absorbance changes of (A) Cys (pH 6.0), (B) Hcy (pH 6.0), (C) Cys (pH 8.8), and (D) Hcy (pH 8.8) solutions after incubation with CB[7] for certain duration and subsequent reaction with DTNB. (E) Percentage complexation of CB[7] with Cys and Hcy at pH 6.0 and 8.8.

The chemical complexation of Hcy with CB[7] was examined using a DTNB assay. DTNB reacts with a thiol group (-SH) and exhibits an absorbance maximum at 412 nm. The DTNB assay involves a reversible reaction that is suitable for host–guest binding studies [7]. After the incubation of Cys with CB[7] (0–30 min; 1:20 ratio) at pH 6.0 and subsequent DTNB addition, the absorbance of the solution at 412 nm decreased. The absorbance decreased gradually with increasing incubation time, suggesting that Cys progressively combined with CB[7]. As the thiol group (-SH) of Cys was blocked by the CB[7] cavity, DTNB could not react with it (Fig. 1A). Meanwhile, after the incubation of Hcy with CB[7] (0–30 min; 1:20 ratio) at pH 6.0 and subsequent DTNB addition, the absorbance at 412 nm did not change (Fig. 1B), suggesting that Hcy did not complex with CB[7] at this pH. In contrast, at pH 8.8, the incubation of Cys with CB[7] (0–30 min; 1:20 ratio) did not lead to an absorbance change at 412 nm for the DTNB-added solution (Fig. 1C), whereas the incubation of Hcy with CB[7] (0–30 min; 1:20 ratio) led to a substantial decrease in the absorbance at 412 nm (Fig. 1D), suggesting that Hcy could efficiently form a supramolecular complex with CB[7]. As a control experiment, the absorbances of iodoacetamide-treated Cys/CB[7] and Hcy/CB[7] solutions were measured after DTNB addition. The absorbance band at 412 nm did not appear because iodoacetamide reacted with the -SH groups. The DTNB assay confirmed that CB[7] could selectively form a supramolecular complex with either Cys or Hcy according to the pH. Fig. 1E compares the absorbances of Cys/CB[7] and Hcy/CB[7] solutions (after DTNB addition) depending on the incubation time at pH 6.0 and 8.8, respectively.

### Investigation of the Supramolecular Complexation by NMR Spectroscopy

**Fig. 2:**
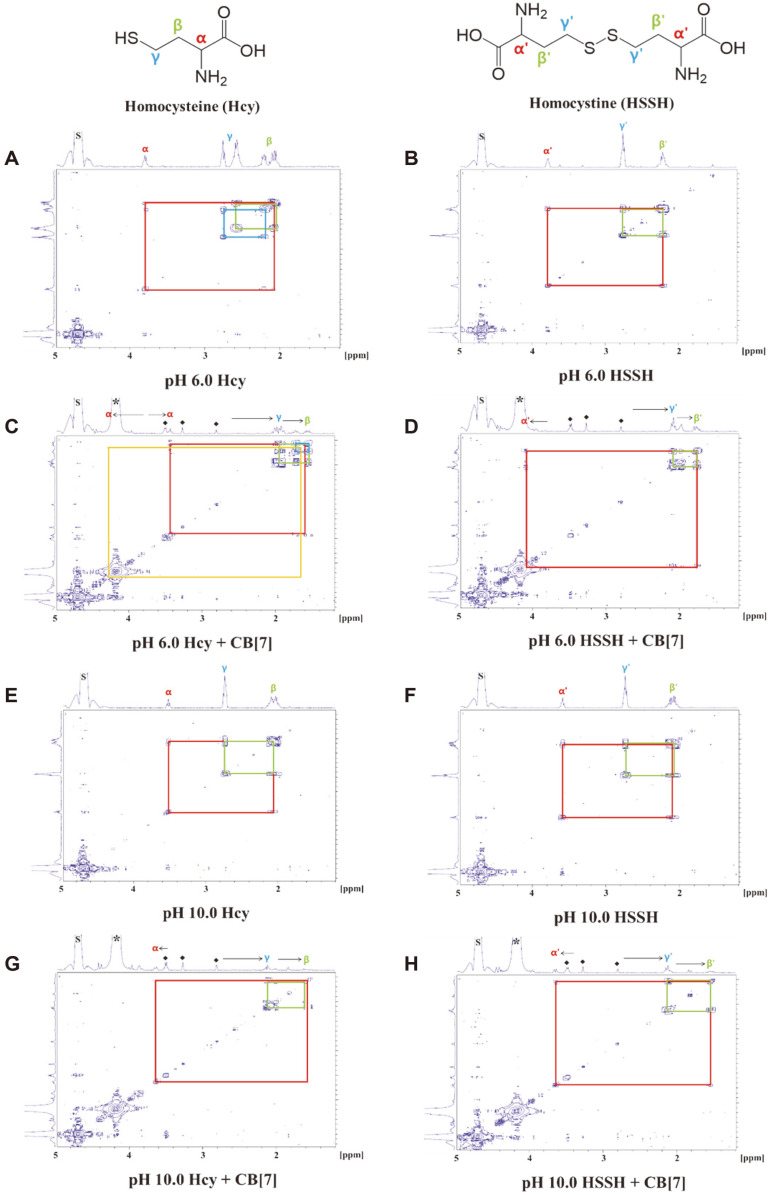
2D NMR (COSY) spectra (400 MHz, D_2_O): Hcy (A) and HSSH (B) in the absence of CB[7], and Hcy (C) and HSSH (D) in the presence of CB[7] at pH 6. Hcy (E) and HSSH (F) in the absence of CB[7], and Hcy (G) and HSSH (H) in the presence of CB[7] at pH 10. The length of the arrow indicates the peak shift. The asterisk (*) and letter (S) denote resonances for CB[7] and undeuterated solvent, respectively. Low-intensity peaks (◆) observed in the NMR spectra of C, D, G, and H are due to unidentified impurities from commercial CB[7].

The supramolecular complexation of the thiols by CB[7] was investigated using ^1^H NMR spectroscopy, including 2D NMR spectroscopy (COSY). Fig. 2 shows the 2D NMR COSY spectra of Hcy and HSSH recorded in the absence and presence of the CB[7] host in D_2_O at pH 6.0 and 10.0.

At pH 6.0, after 36 h of incubation under ambient conditions, the Hcy solution provided two sets of β and γ proton resonances (Fig. 2A), among which one set of β and γ proton resonances was very similar to that of HSSH, as determined by comparison with the NMR spectrum of HSSH at pH 6.0 (Fig. 2B). This result suggests that a fraction of Hcy was spontaneously oxidized to HSSH when Hcy was incubated alone at pH 6.0 for 36 h. However, in the presence of CB[7] at pH 6.0, the ethylene proton peaks of Hcy (δ in ppm = 1.90 for H_γ_ and 1.55–1.75 for H_β_) displayed a substantial upfield shift compared with those of Hcy alone (δ in ppm = 2.55–2.75 for H_γ_ and 2.00–2.20 for H_β_) (Fig. 2C), indicating that the hydrophobic cavity of CB[7] provides a shielding environment for the ethylene protons of Hcy. The signal corresponding to the α proton of Hcy (H_α_) was also shifted to a higher field (Δδ = −0.37 ppm), indicating that the guest molecule, Hcy, was included in the cavity of the host, CB[7] [15]. Thus, it was inferred that the supramolecular complexes of Hcy-CB[7] and HSSH-CB[7] coexist under acidic conditions after 36 h of incubating Hcy with CB[7] (Fig. 2C).

**Fig. 3:**
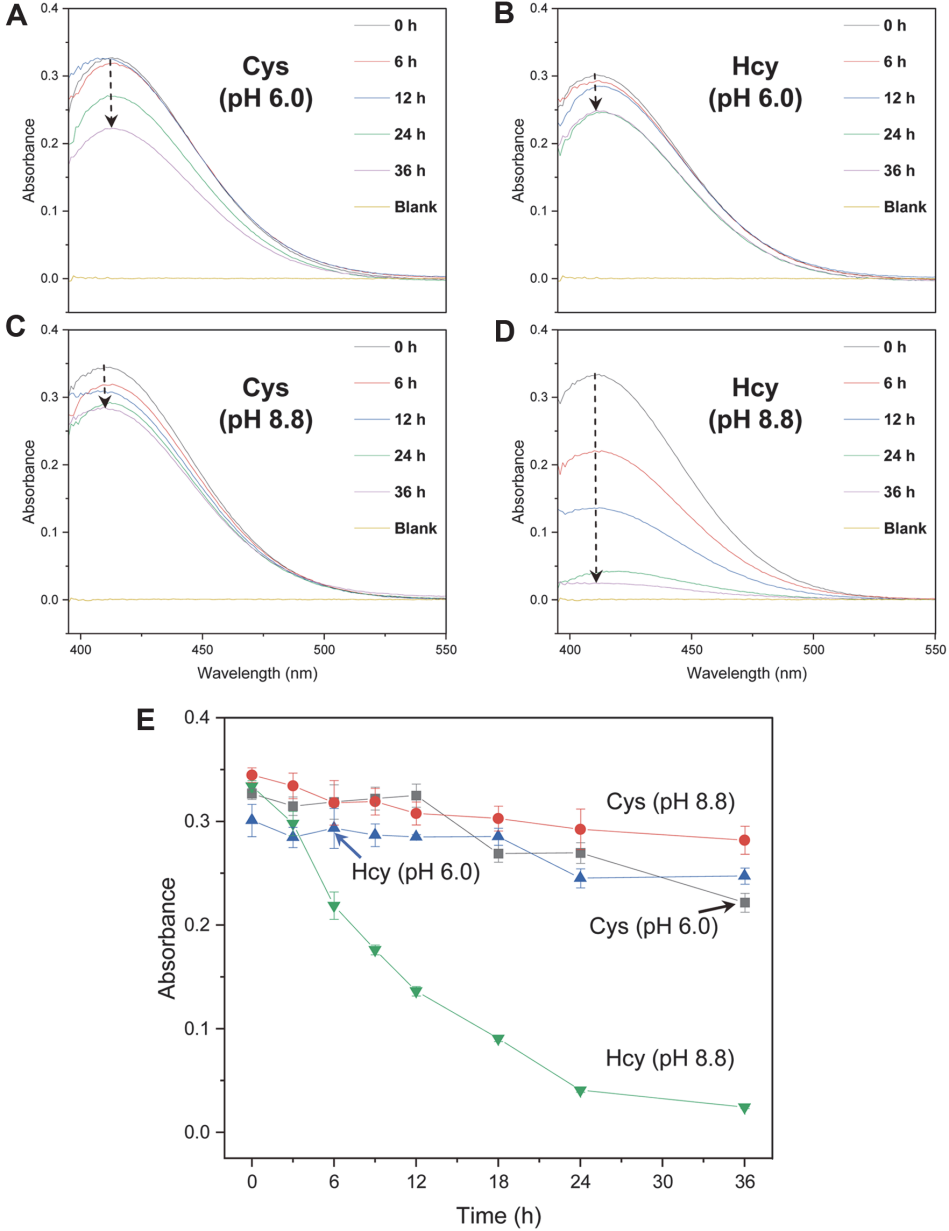
Absorbance spectral changes of (A, C) Cys and (B, D) Hcy solutions incubated at the indicated pH for 0 to 36 h and then reacted with DTNB. (E) Changes in the absorbance at 412 nm with time.

**Fig. 4:**
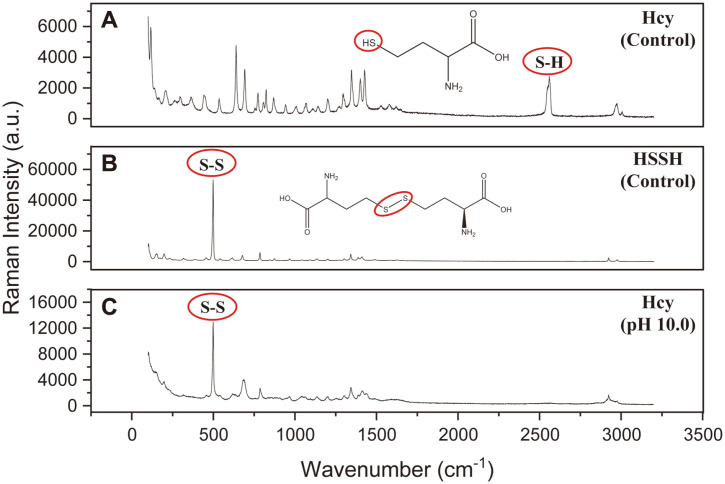
Raman spectra of controls (A) Hcy, (B) HSSH and after 36 h (C) Hcy in pH 10.0 aqueous conditions, SH group disappeared and S-S group was observed. The S-H bond (2,500-2,600 cm^-1^) and S-S bond (450-550 cm^-1^) were labeled as circles.

At pH 10.0, the 2D NMR COSY spectrum of Hcy incubated for 36 h under ambient conditions showed a single set of three signals located at δ (ppm) of 3.51 (H_α_), 2.74 (H_γ_), and 2.04 (H_β_) (Fig. 2E). These signals were almost identical to those observed for HSSH, δ (ppm) = 3.59, 2.74, and 2.09 (Fig. 2F). These results suggest that Hcy auto-oxidized to HSSH under basic aqueous conditions in the absence of CB[7], which was additionally confirmed by the DTNB assay (Fig. 3) and Raman spectroscopy (Fig. 4). The 2D NMR spectra of the Hcy-CB[7] (Fig. 2G) and HSSH-CB[7] complexes (Fig. 2H) also exhibited very similar patterns at pH 10.0.

In the presence of CB[7], the resonances for the β’ and γ’ protons of HSSH were shifted upfield (Figs. 2D and 2H) under both acidic and basic conditions. These results imply that the β’ and γ’ protons entered the cavity of the CB[7] and experienced a shielding effect. In contrast, the α’ proton peak of HSSH underwent a downfield shift because it protruded out of the CB[7] cavity due to its longer chain length compared with the cavity depth of CB[7](Figs. 2D and 2H). Our data indicate that HSSH can form a supramolecular complex with CB[7] and that the α proton of the Hcy-CB[7] complex is located inside the CB[7] cavity, whereas the α’ proton of the HSSH-CB[7] complex is located outside the cavity.

Meanwhile, 2D NMR data suggested that Cys-CB[7] complexation occurred at pH 6.0, but not at pH 10.0. The CSSC-CB[7] complexation was not detected at pH 6.0 or 10.0 (data not shown). According to the 2D NMR results, Hcy oxidizes to HSSH under basic conditions and efficiently forms a supramolecular complex with CB[7].

### Autoxidation of Cys and Hcy

Investigations by 2D NMR spectroscopy revealed that Hcy was oxidized to HSSH, resulting in efficient supramolecular complexation with CB[7] under basic pH conditions. Therefore, we used the DTNB method to study the time-dependent autoxidation of Cys and Hcy under acidic and basic pH in aqueous solutions in the absence of CB[7]. After 36 h of incubation, the absorbances of Cys (pH 6.0), Cys (pH 8.8), and Hcy (pH 6.0) solutions mixed subsequently with DTNB decreased by up to 32.14, 17.82, and 18.20% (Figs. 3A-3C). Meanwhile, the absorbance of Hcy incubated at pH 8.8 for 36 h and then mixed with DTNB decreased by up to 92.74%(Figs. 3D and 3E). The decrease in the absorbance of the solution suggests the loss of free SH groups and implies that Hcy underwent autoxidation to HSSH at the basic pH. The rate of Hcy oxidation to HSSH was much faster in the presence of CB[7] than in the absence of CB[7] (Figs. 1D and 3D).

We further verified whether Hcy was auto-oxidized in a basic pH aqueous solution via Raman spectroscopy, a method that helps identify specific atoms in a chemical bond based on the vibrational energy. Initially, Hcy yielded a distinct Raman peak for the S-H bond (2,500–2,600 cm^−1^) (Fig. 4A). After 36 h of incubation under basic conditions, the signal of the S-H bond disappeared and a new signal of the S-S bond appeared (450–550 cm^−1^) in the Raman spectrum (Fig. 4C). Moreover, the Raman spectrum of Hcy obtained after 36 h of incubation under basic conditions was similar to that of HSSH (Figs. 4B and 4C). On the other hand, the Raman spectrum of Cys did not change significantly under basic conditions after 36 h of incubation (data not shown).

## Discussion

**Fig. 5:**
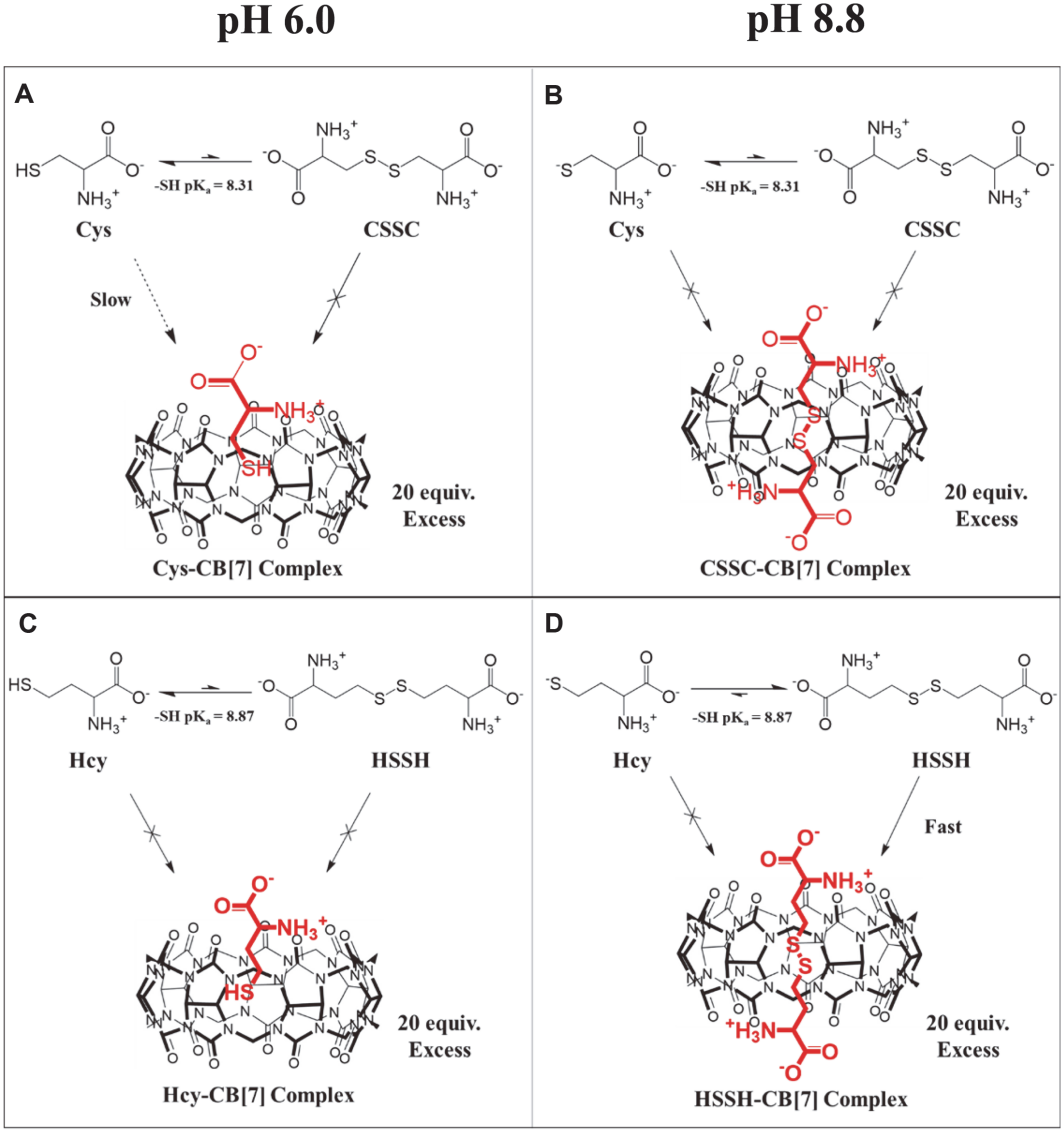
Proposed supramolecular complexation of (A-B) Cys and (C-D) Hcy with CB[7] at the indicated pH.

This is the first report on the selective complexation of HSSH over Hcy with CB[7] under basic pH conditions (pH 8.8~10.0). We explored the supramolecular complexation of the CB[7] host with two biothiol guests, Cys and Hcy, in buffer solutions of different pH. Based on the results of the DTNB assay and 2D NMR investigations, we propose a supramolecular model for the complexation of Cys and Hcy with CB[7] depending on the pH condition (Fig. 5). According to the DTNB assay, at pH 6.0, up to 28.92% of Cys binds to CB[7] depending on the incubation time (Figs. 1A and 5A). Although the formation of Hcy-CB[7] and HSSH-CB[7] complexes was indicated by 2D NMR spectra obtained after 36 h of incubation (Fig. 2C), Hcy did not bind to CB[7] within 30 min of incubation (Figs. 1B and 5C). On the other hand, at pH 8.8, when Hcy was incubated with CB[7] for 15 min, more than 90% of the ethyl thiol group of Hcy was buried in the cavity of CB[7] (Figs. 1D and 5D). In contrast, Cys was not included in CB[7] in the buffer solutions; it either existed freely or the methylene thiol group was outside the CB[7] cavity when the ammonium group of Cys interacted with the carbonyl portal of CB[7]. Therefore, the DTNB reagent could fully recognize the methylene thiol group of Cys under basic conditions (Figs. 1C and 5B).

The turnover rate of the oxidation of Hcy to HSSH in the presence of CB[7] under basic conditions was 150 times faster than that in the absence of CB[7] (Figs. 1D and 3D). In general, the chemical equilibrium between Hcy and HSSH changes dynamically with the concentration of the chemical species in the solution. Therefore, the equilibrium between Hcy and HSSH in the presence of CB[7] is thought to shift toward HSSH as free HSSH is depleted owing to host–guest complex formation (Fig. 5D). As the Hcy-CB[7] complex could not be detected under basic conditions within 30 min of incubation, the host CB[7] probably formed a thermodynamically stable supramolecular complex of HSSH-CB[7] under basic conditions. We also speculate that the CB[7] cavity served as a reaction chamber for the reaction between two Hcy species that entered from opposite portals to form HSSH. Consequently, CB[7] substantially enhanced the oxidation of Hcy to HSSH. In fact, reactions catalyzed by CB have been reported, and some examples include the formation of oligotriazoles, acidic decomposition of azidoaminoalkanes, and epoxide alcoholysis [26-28]. Moreover, upon the formation of the HSSH-CB[7] complex, the positively charged ammonium group (NH_3_^+^) (pKa = 9.44) of HSSH stabilizes the complex through attractive interaction with the carbonyl portal of CB[7], and the carboxylate group of HSSH is located outside the CB[7] cavity owing to electrostatic repulsion between the anionic carboxylate group and the carbonyl oxygen atoms of the CB[7] (Fig. 5D). Given the depth of the CB[*n*] cavity (9.1 Å), complexes of protonated diamines with five or six methylene groups were reported to have the highest stability (*K*_CB[6]_ = 2–3 × 10^8^ M^−1^) [29]. Therefore, the hydrophobic -CH_2_-CH_2_-S-S-CH_2_-CH_2_- chain of HSSH, which fits well into the CB[7] cavity, critically contributes to the formation of a stable HSSH-CB[7] complex.

On the other hand, Cys, which has a low pK_a_ and lacks a methylene unit compared to Hcy, could hardly be included in the cavity of CB[7]. As the total concentration of Hcy in the plasma or serum is approximately 5 to 10 μM, host–guest complexation with CB[7] will be useful in detecting the cardiovascular risk factor, Hcy concentration, in plasma or serum samples.

## References

[1] Wood ZA, Schröder E, Robin Harris J, Poole LB (2003). Structure, mechanism and regulation of peroxiredoxins. Trends Biochem. Sci..

[2] Yin G, Niu T, Yu T, Gan Y, Sun X, Yin P (2019). Simultaneous visualization of endogenous homocysteine, cysteine, glutathione, and their transformation through different fluorescence channels. Angew. Chem. Int. Ed. Engl..

[3] Clarke R, Smith AD, Jobst KA, Refsum H, Sutton L, Ueland PM (1998). Folate, Vitamin B12, and serum total homocysteine levels in confirmed alzheimer disease. Arch. Neurol..

[4] Nygård O, Vollset SE, Refsum H, Cappelen I, Tverdal A, Nordrehaug J (1995). Total plasma homocysteine and cardiovascular risk profile. The hordaland homocysteine study. JAMA.

[5] Page JH, Ma J, Chiuve SE, Stampfer MJ, Selhub J, Manson JE (2010). Plasma total cysteine and total homocysteine and risk of myocardial infarction in women: a prospective study. Am. Heart J..

[6] Ueland PM, Refsum H, Beresford SA, Vollset SE (2000). The controversy over homocysteine and cardiovascular risk. Am. J. Clin. Nutr..

[7] Park S-H, Lee J-Y, Cho H-N, Kim K-R, Yang S-A, Kim H-J (2019). Simple and novel assay of the host-guest complexation of homocysteine with Cucurbit[7]uril. J. Microbiol. Biotechnol..

[8] Inoue T, Kirchhoff JR (2002). Determination of thiols by capillary electrophoresis with amperometric eetection at a coenzyme pyrroloquinoline quinone modified electrode. Anal. Chem..

[9] Wang W, Rusin O, Xu X, Kim KK, Escobedo JO, Fakayode SO (2005). Detection of homocysteine and cysteine. J. Am. Chem. Soc..

[10] Rusin O, St. Luce NN, Agbaria RA, Escobedo JO, Jiang S, Warner IM (2004). Visual detection of cysteine and homocysteine. J. Am. Chem. Soc..

[11] Wang J, Liu Y, Jiang M, Li Y, Xia L, Wu P (2018). Aldehyde-functionalized metal-organic frameworks for selective sensing of homocysteine over Cys, GSH and other natural amino acids. Chem. Commun..

[12] Niu L-Y, Chen Y-Z, Zheng H-R, Wu L-Z, Tung C-H, Yang Q-Z (2015). Design strategies of fluorescent probes for selective detection among biothiols. Chem. Soc. Rev..

[13] Fan W, Huang X, Shi X, Wang Z, Lu Z, Fan C (2017). A simple fluorescent probe for sensing cysteine over homocysteine and glutathione based on PET. Spectrochim. Acta A Mol. Biomol. Spectrosc..

[14] Wang W, Li L, Liu S, Ma C, Zhang S (2008). Determination of physiological thiols by electrochemical detection with piazselenole and its application in rat breast cancer cells 4T-1. J. Am. Chem. Soc..

[15] Lee MJ, Shee NK, Son J-I, Karthikeyan S, Jhee K-H, Lee JY (2019). Supramolecular complexation of homocysteine and cysteine with cucurbit[7]uril. Supramol. Chem..

[16] Li J, Loh X (2008). Cyclodextrin-based supramolecular architectures: syntheses, structures, and applications for drug and gene delivery. Adv. Drug Del. Rev..

[17] Busschaert N, Caltagirone C, Van Rossom W, Gale PA (2015). Applications of supramolecular anion recognition. Chem. Rev..

[18] Barrow SJ, Kasera S, Rowland MJ, Del Barrio J, Scherman OA (2015). Cucurbituril-based molecular recognition. Chem. Rev..

[19] Biedermann F, Nau WM (2014). Noncovalent chirality sensing ensembles for the detection and reaction monitoring of amino acids, peptides, proteins, and aromatic drugs. Angew. Chem. Int. Ed. Engl..

[20] Gao Z-Z, Lin R-L, Bai D, Tao Z, Liu J-X, Xiao X (2017). Host-guest complexation of cucurbit[8]uril with two enantiomers. Sci. Rep..

[21] Freeman WA, Mock WL, Shih NY (1981). Cucurbituril. J. Am. Chem. Soc..

[22] Masson E, Ling X, Joseph R, Kyeremeh-Mensah L, Lu X (2012). Cucurbituril chemistry: a tale of supramolecular success. RSC Adv..

[23] Urbach AR, Ramalingam V (2011). Molecular recognition of amino acids, peptides, and proteins by Cucurbit[n]uril receptors. Isr. J. Chem..

[24] Ahmed KA, Sawa T, Akaike T (2011). Protein cysteine S-guanylation and electrophilic signal transduction by endogenous nitronucleotides. Amino Acids.

[25] Iciek M, Chwatko G, Lorenc-Koci E, Bald E, Włodek L (2004). Plasma levels of total, free and protein bound thiols as well as sulfane sulfur in different age groups of rats. Acta Biochim. Pol..

[26] Krasia TC, Steinke JH (2002). Formation of oligotriazoles catalysed by cucurbituril. Chem. Commun..

[27] Wieland M, Mieusset J-L, Brinker UH (2012). Cucurbit [6] uril as a potential catalyst for the acidic decomposition of azidoaminoalkanes. Tetrahedron Lett..

[28] Xu L, Fang G, Yu Y, Ma Y, Ye Z, Li Z (2019). Molecular mechanism of heterogeneous supramolecular catalysis of metal-free cucurbituril solid for epoxide alcoholysis. Mol. Catal..

[29] Mock WL, Shih NY (1986). Structure and selectivity in host-guest complexes of cucurbituril. J. Org. Chem..

